# Determinants of Maximal Dorsiflexion Range of Motion: Multi-Perspective Comparison Using Mechanical, Neural, Morphological, and Muscle Quality Factors

**DOI:** 10.3390/jfmk9040257

**Published:** 2024-12-05

**Authors:** Takamasa Mizuno, Akito Yoshiko, Naoyuki Yamashita, Kenji Harada, Kosuke Takeuchi, Shingo Matsuo, Masatoshi Nakamura

**Affiliations:** 1 Research Center of Health, Physical Fitness and Sports, Nagoya University, Nagoya-shi 464-8601, Aichi, Japan; 2 Faculty of Liberal Arts and Sciences, Chukyo University, Toyota-shi 470-0348, Aichi, Japan; yoshiko@lets.chukyo-u.ac.jp; 3 Faculty of Arts and Sciences, Kyoto Institute of Technology, Kyoto-fu 606-0951, Kyoto, Japan; ynaoyuki@kit.ac.jp; 4 Department of Preventive Gerontology, Center for Gerontology and Social Science, National Center for Geriatrics and Gerontology, Obu-shi 474-8511, Aichi, Japan; knj.harada@gmail.com; 5 Department of Physical Therapy, Kobe International University, Kobe-shi 658-0032, Hyogo, Japan; ktakeuchi@kobe-kiu.ac.jp; 6 Department of Rehabilitation, Faculty of Health Sciences, Nihon Fukushi University, Handa-shi 475-0012, Aichi, Japan; matsuo@n-fukushi.ac.jp; 7 Faculty of Rehabilitation Sciences, Nishi Kyushu University, Kanzaki-shi 842-0015, Saga, Japan; nakamuramas@nisikyu-u.ac.jp

**Keywords:** echo intensity, passive torque, stepwise multiple regression analysis, stretch tolerance, ultrasound

## Abstract

**Background/Objectives**: the purpose of this study was to determine the contributions of mechanical, neural, morphological, and muscle quality factors on individual differences in the maximal ankle dorsiflexion range of motion (ROM). **Methods**: A sample of 41 university students performed passive-dorsiflexion and morphological measurements. In the passive-dorsiflexion measurement, while the ankle was passively dorsiflexed, maximal dorsiflexion ROM was measured in addition to passive torque at a given angle and muscle–tendon junction (MTJ) displacement during the last 13° as mechanical factors, and stretch tolerance and muscle activation were measured as neural factors. In morphological measurements, the cross-sectional area, muscle thickness, muscle fascicle length, and pennation angle were measured. In addition, the echo intensity was evaluated as muscle quality. Subjects were divided into three groups (flexible, moderate, and tight) using the value of the maximal dorsiflexion ROM. **Results**: Maximal dorsiflexion ROM and stretch tolerance were greater in the flexible group than those in the moderate and tight groups. MTJ displacement was smaller in the flexible group than those in the moderate and tight groups. Stepwise multiple regression analysis revealed that stretch tolerance and passive torque at a given angle were selected as parameters to explain the maximal dorsiflexion ROM (adjusted *R*^2^ = 0.83). **Conclusions**: these results indicate that individual differences in maximal ankle dorsiflexion ROM are primarily related to mechanical and neural factors.

## 1. Introduction

The maximal joint range of motion (ROM) is often used as a representative index of joint flexibility. Additionally, maximal joint ROM has also been reported to be associated with the isometric torque–angle relationship [1] and activities of daily living [2]. Other studies have reported that greater maximal joint ROM improves exercise performance, such as dancing [3], and decreases injury risk [4]. Thus, achieving optimal or greater joint ROM is beneficial for activities of daily living and exercise. However, large individual differences have been reported in maximal joint ROM [1,5]. Hence, identifying the factors that determine an individual’s maximal ROM may allow for the improvement of daily activities, physical performance, and injury prevention in various populations [2,3,4]. Although multiple factors have been reported to be associated with individual differences in maximal joint ROM, including mechanical, neural, and muscle morphological factors [5,6,7,8], the relative importance among these factors is currently not fully understood.

Previous studies have examined the influences of mechanical factors of the muscle–tendon unit, neural factors, and muscle morphological factors on maximal joint ROM [5,6,7,8]. Blazevich et al. [5] reported that the greater maximal dorsiflexion ROM group showed less passive torque at 30° of dorsiflexion, greater passive torque at the maximal dorsiflexed position (i.e., greater stretch tolerance), and greater lengthening of muscle and tendon from 20° of dorsiflexion to maximal dorsiflexion angle compared with the lesser maximal dorsiflexion ROM group. No differences in peak electromyography amplitudes in the soleus and gastrocnemius medialis muscles were found during passive ankle dorsiflexion between the groups [5]. Furthermore, significant correlations were found between maximal ankle dorsiflexion ROM and stretch tolerance, fascicle rotation measured from the neutral ankle angle to 30° of dorsiflexion, and the angle of electromyography onset [5]. The authors noted that significant differences in neuromuscular responses to ankle dorsiflexion depended on the dorsiflexion ROM [5]. In contrast, Suga et al. [8] reported a significant correlation between maximal dorsiflexion ROM and muscle thickness, muscle volume, and muscle cross-sectional area of the plantar flexor muscles in young men. However, none of these previous studies fully considered the combined influences of these factors (consisting of mechanical, neural, and morphological factors) on maximal dorsiflexion ROM [5,8]. A study by Moltubakk et al. [7] compared professional ballet dancers with active control subjects by comparing mechanical, neural, and morphological factors. They concluded that the greater maximal dorsiflexion ROM of ballet dancers can be explained by a combination of factors, including muscle and tendon morphology, mechanical factors of the plantar flexion muscle, and neural activity. Magnusson et al. [6] divided 18 male elite-level orienteers into tight and normal groups on the basis of toe-touch test results and compared differences in mechanical, neural, and morphological factors. The tight group had a lower maximal knee extension angle than the normal group, as well as lower stretch tolerance, but there was no difference in the cross-sectional area of the biceps femoris muscle [6]. However, the greater joint ROM in ballet dancers and orienteers was likely to have been acquired through ballet dance or orienteering training. Hence, it is uncertain whether the mechanical, neural, and morphological characteristics of ankle ROM observed in these athletes would also be observed in an untrained population.

Muscle quality may also affect individual differences in maximal joint ROM. Echo intensity calculated using the black-to-white color scale from B-mode ultrasound images has been used as an indicator of muscle quality in previous studies [9,10]. Echo intensity is significantly associated with non-contractile elements such as intramuscular fat and/or connective tissue [11,12,13]. Muscle fat concentration and connective tissue are some of the limiting factors of maximal ROM [14]. Thus, echo intensity could be one of the limiting factors for maximal joint ROM, but the relationship between echo intensity and maximal joint ROM has not been clarified to date.

As described above, multiple factors are thought to be involved in maximal joint ROM. However, to the best of our knowledge, it is unclear whether complex factors are involved in individual differences in maximal joint ROM in the general population, which is not composed solely of specific competitive athletes, such as dancers. To further clarify the difference between the flexible and tight ROM groups, subjects were divided into three groups according to the maximal dorsiflexion ROM. We hypothesized that a combination of mechanical, neural, morphological, and muscle quality factors are related to individual differences in maximal dorsiflexion ROM; however, it is unclear which factors are involved in maximal dorsiflexion ROM. To test this hypothesis, the current study sought to determine the contributions of mechanical, neural, morphological, and muscle quality factors on individual differences in maximal dorsiflexion ROM. Multiple regression analysis was also used to detect the contribution of these factors to maximal dorsiflexion ROM. If the main factors that influence individual differences in maximal dorsiflexion ROM can be clarified from various perspectives in the current study, these findings may be helpful for designing targeted interventions to improve maximal dorsiflexion ROM. The establishment of such methods for increasing maximal joint ROM not only could contribute to athletic performance, such as improving performance in dance and gymnastics [3], in which flexibility plays an important role in the performance and prevention of injury during exercise [15,16], but also could be helpful in rehabilitation and daily function, such as improving balance, gait, lower limb mobility, and fall risk [17,18,19,20].

## 2. Materials and Methods

### 2.1. Subjects

Forty-two young men and women volunteered for this study, and the final cohort consisted of 41 men and women (20 men [age: 20.6 ± 1.5 years; height: 172.7 ± 8.8 cm; weight: 67.8 ± 17.4 kg; and exercise time: 132.5 ± 158.9 min/week] and 21 women [age: 19.8 ± 1.0 years; height: 158.9 ± 6.0 cm; weight: 51.2 ± 8.6 kg; and exercise time: 186.9 ± 178.0 min/week]). One subject was excluded on the basis of the Smirnov–Grubbs rejection test (*p* < 0.05) because the value of maximal dorsiflexion ROM was too high (outlier: 42.23 and overall: 15.2 ± 10.3). Thirteen subjects (six men and seven women) had not exercised in at least the last month. Twenty-five subjects (12 men and 13 women) were recreationally active, mainly engaging in walking, jogging, running, strength training, dance, and tennis. Three subjects (two men and a woman) participated in university extracurricular activities in kyudo (Japanese archery), karate, and sumo. Thus, unlike previous studies [6,7], the subjects in this study were not a group of individuals who played the same competitive sport. None of the subjects had a history of recent musculoskeletal injury or neuromuscular disease specific to the lower limb. Subjects provided written informed consent for their participation in the experiments, which were conducted according to the principles of the Declaration of Helsinki. All subjects were fully informed of the purposes, procedures, and possible risks of the study. The experimental protocol was approved by the local ethical committee.

### 2.2. Morphological Measurement

**Figure 1:**
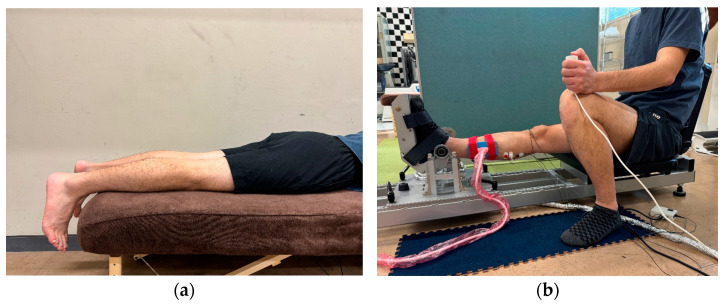
Posture during morphological and passive-dorsiflexion measurements. (**a**) Subjects were resting in a prone position with their feet dangling freely from the examination bed during morphological measurement. (**b**) The right foot of the subject was secured to an isokinetic machine with the right knee fully extended during passive-dorsiflexion measurement. The back seat was angled at 75° to the floor.

Muscle morphology and echo intensity were measured using the following previous studies [10,21,22]. Muscle thickness, fascicle length, and pennation angle of the gastrocnemius medialis, as well as the cross-sectional area of the gastrocnemius muscle (i.e., gastrocnemius medialis and gastrocnemius lateralis), were measured in the right lower limb using an ultrasound imaging device (Versana Active; GE Healthcare Japan, Tokyo, Japan) and a linear array probe (12L-RS; GE Healthcare Japan, Tokyo, Japan). The acquisition parameters were as follows: frequency, 10 MHz; gain, 63 dB; depth, 4.0 cm; and focus point, top of the image. A water-soluble gel was applied to the scanning head of the probe to achieve acoustic coupling. Subjects were resting in a prone position with their feet dangling freely from the examination bed (Figure 1a). The angle of the right lower limb between the line drawn from the fifth metatarsal to the heel and the line drawn from the external capsule to the fibular head was adjusted with a manual angle meter (MMI goniometer; Muranaka Medical Instruments Co., Ltd., Osaka, Japan) to ensure that the angle was perpendicular.

**Figure 2:**
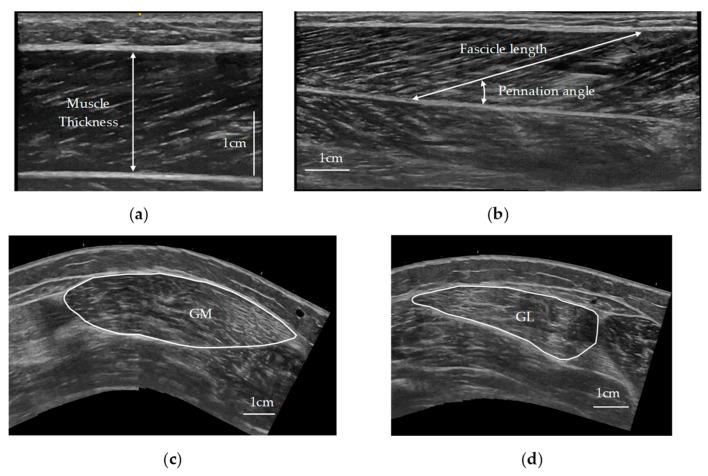
Measurements of muscle thickness, fascicle length, pennation angle, cross-sectional area, and echo intensity. (**a**) Muscle thickness was defined as the perpendicular distance between the superficial and deep aponeuroses of the gastrocnemius medialis muscle. (**b**) Fascicle length was defined as a clearly visible fiber bundle near the center of the ultrasound panorama image lying between the superficial and deep aponeuroses of the gastrocnemius medialis. The pennation angle was defined as a clearly visible angle near the center of the ultrasound panorama image formed between the deep aponeurosis and a muscle fascicle. (**c**,**d**) The solid line outlines cross-sectional area of the gastrocnemius medialis muscle (GM) and the gastrocnemius lateralis muscle (GL). The GM and GL were traced on the basis of the surface fascia and the upper boundary of the deep fascia. The mean echo intensity within the muscle was calculated for each image.

Muscle thickness of the gastrocnemius medialis was measured from sagittal plane ultrasound images at the proximal 30% and 40% of the lower-leg length. Lower-leg length was defined as the distance from the popliteal fossa to the lateral malleolus of the fibula. The mean value of the measurements taken at 30% and 40% of the lower-leg length was used for subsequent analyses. Muscle thickness was defined as the perpendicular distance between the superficial and deep aponeuroses of the gastrocnemius medialis (Figure 2a).

Fascicle length and pennation angle of gastrocnemius medialis were measured from sagittal plane ultrasound panorama images (LOGIQ View; GE Healthcare Japan, Tokyo, Japan) from the proximal 20% to 50% of the length of the lower leg. Fascicle length was defined as a clearly visible fiber bundle near the center of the ultrasound panorama image lying between the superficial and deep aponeuroses of the gastrocnemius medialis (Figure 2b). The pennation angle was defined as a clearly visible angle near the center of the ultrasound panorama image formed between the deep aponeurosis and a muscle fascicle (Figure 2b). Three fascicle lengths and three pennation angles were measured, and the average of each was used for subsequent analysis.

The cross-sectional area of the gastrocnemius muscle was measured from horizontal plane ultrasound panorama images (LOGIQ View) at the proximal 30% and 40% of the lower-leg length (Figure 2c,d). The values of cross-sectional area for gastrocnemius medialis and gastrocnemius lateralis were summed at 30% and 40% of the lower-leg length, respectively. The mean value of the measurements taken at 30% and 40% of the lower-leg length was used for subsequent analyses.

The ultrasound probe was positioned at the thickest portion of the gastrocnemius medialis in the measurement of the muscle thickness, fascicle length, and pennation angle. The images were stored in the Digital Imaging and Communications in Medicine format. Muscle architecture parameters of ultrasonography were analyzed using ImageJ (ImageJ 1.50i; NIH, Bethesda, MD, USA).

Echo intensity was evaluated on the basis of 256 grayscale levels per pixel using ImageJ software and expressed in arbitrary units (a. u.). Echo intensity was evaluated using horizontal plane images at proximal 30% and 40% positions in gastrocnemius medialis and gastrocnemius lateralis. The mean echo intensity within the muscle was calculated for each image (Figure 2c,d). Echo intensities evaluated at the 30% and 40% positions in gastrocnemius medialis and gastrocnemius lateralis, respectively, were averaged, and the echo intensities of gastrocnemius medialis and gastrocnemius lateralis were then averaged as the echo intensity for subsequent analyses.

### 2.3. Passive-Dorsiflexion Measurement

**Figure 3:**
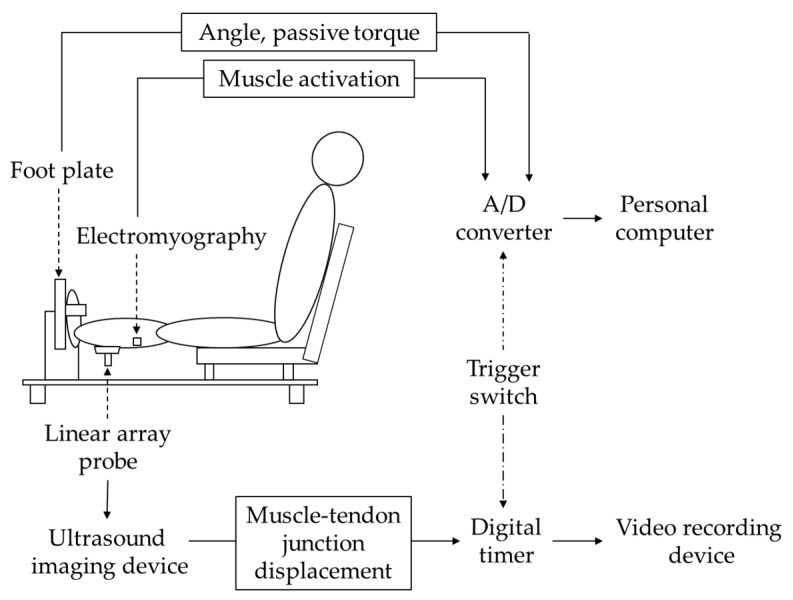
Schematic diagram of the experimental set-up in a passive-dorsiflexion measurement. Ankle angle and passive torque were converted from analog to digital values at a sampling rate of 1 kHz. The electromyography signals were transmitted to a digital data recorder at a sampling rate of 1.0 kHz and recorded at a 5–500 Hz bandwidth. These data were recorded on a personal computer. The muscle–tendon junction images were recorded on a video recording device via a digital timer and synchronized with ankle angle and passive torque output by activating the trigger switch.

To determine the maximal dorsiflexion ROM, mechanical properties such as passive torque and displacement of muscle–tendon junction (MTJ), and neural properties such as stretch tolerance and electromyography amplitude were measured; each subject underwent passive-dorsiflexion measurement, which was performed in the same way as that reported in previous studies [23,24,25]. The right foot of the subject was secured to an isokinetic machine (S-15177; Takei Scientific Instruments, Niigata, Japan) with the right knee fully extended (Figure 1b). The back seat was angled at 75° to the floor. In this study, the footplate angle is shown as the ankle joint angle, which is defined as 0° when the footplate is perpendicular to the floor. Dorsiflexion is indicated by positive values and plantar flexion by negative values. The subject’s ankle joint was passively dorsiflexed at a rate of 1°/s from −30°. Dorsiflexion was stopped by pressing a switch when the subject felt discomfort (i.e., onset of pain) in the lower limb, and the angle at that point was used as the maximal dorsiflexion ROM. The passive torque generated on the footplate was evaluated at −4° and the maximal dorsiflexed position. The passive torque at −4° was the maximal dorsiflexion angle reached by all subjects during the passive-dorsiflexion measurement and was used to compare the passive torque at the same joint angle among subjects. The passive torque at the maximal dorsiflexed position was used as an index of stretch tolerance. During the passive-dorsiflexion measurement, subjects were asked to be completely relaxed and not to offer any voluntary contraction. Passive torque and ankle angle were converted from analog to digital values at a sampling rate of 1.0 kHz (PowerLab 16SP; PowerLab System, AD Instruments Pty Ltd., Bella Vista, NSW, Australia) (Figure 3). The measurement that recorded the greater maximal dorsiflexion ROM during the two passive-dorsiflexion measurements was used for all subsequent analyses.

Simultaneously, the displacement of the MTJ of the gastrocnemius medialis was determined using B-mode ultrasonography (Versana Active) during the passive-dorsiflexion measurement. The linear array probe (12L-RS) was fixed to the skin. The MTJ was visualized as a longitudinal ultrasound image and synchronized with passive torque and ankle angle output. The relative displacement between the reflective marker affixed on the skin and the MTJ was measured (see [23] in detail). MTJ displacement was evaluated by the amount of displacement in the last 13° during passive dorsiflexion [25].

### 2.4. Electromyography

Muscle activation during passive dorsiflexion in passive-dorsiflexion measurement was measured using electromyography (DL-140; S&ME, Tokyo, Japan). Disposable surface electrodes (F-150s; Nihon Kohden, Tokyo, Japan) were affixed to the gastrocnemius medialis and tibialis anterior muscle at a distance of 20 mm between electrodes. The electrode location was the most prominent bulge of the gastrocnemius medialis and one-third of the distance from the tip of the fibula to the medial malleolus in accordance with Surface Electromyography for the Noninvasive Assessment of Muscle (SENIAM) guidelines. The electromyography signals were transmitted to a digital data recorder at a sampling rate of 1.0 kHz and recorded at a bandwidth of 5–500 Hz. The root mean square was calculated for the last 5° interval from an angle of 5° less than the maximal dorsiflexion position to the maximal dorsiflexion position. Electromyography amplitudes were normalized to the peak electromyography amplitude measured during isometric contractions at 0° to allow for inter-subject comparison. Peak electromyography amplitude during isometric contraction was measured after all measurements in this study.

### 2.5. Data Reliability

The intraclass correlation coefficients for maximal dorsiflexion ROM, stretch tolerance, displacement of MTJ at maximal dorsiflexion position, muscle thickness, and pennation angle were 0.987, 0.975, 0.841, 0.969, and 0.949, respectively.

### 2.6. Statistical Analyses

The electromyography data were available for only 40 of the 41 subjects because electromyography data were not recorded for one subject. All other analyses were conducted using data from all 41 subjects. All statistical analyses were performed using SPSS version 22.0 (IBM Corp., Armonk, NY, USA). Subjects were divided into three groups (flexible, moderate, or tight) on the basis of their maximal dorsiflexion ROM using Ward’s method in hierarchical cluster analysis to avoid arbitrary criteria. Differences among groups in the number of men and women were assessed using the chi-square test. The Shapiro–Wilk test was used to assess normal distribution. Maximal dorsiflexion ROM, height, displacement of the MTJ, cross-sectional area, pennation angle, fascicle length, and echo intensity were normally distributed. The differences among groups for these parameters were assessed using a one-way analysis of variance followed by Bonferroni post hoc test where appropriate. Age, weight, exercise time, passive torque at −4°, stretch tolerance, electromyography, and muscle thickness were not normally distributed, so the Kruskal–Wallis test was used to evaluate the group differences. When appropriate, follow-up analyses were performed using the Mann–Whitney U test with Bonferroni correction. Pearson’s correlation and Spearman’s rank correlation coefficient were calculated to clarify the relationships between maximal dorsiflexion ROM and variables, on the basis of the Shapiro–Wilk test. In addition, stepwise multiple regression analysis was performed with maximal dorsiflexion ROM as the dependent variable and passive torque at −4°, displacement of MTJ, stretch tolerance, electromyography, muscle thickness, cross-sectional area, pennation angle, fascicle length, and echo intensity as independent variables. The multiple regression analysis was performed by including overall participants, and men-only and women-only models. A variance inflation factor of <10 was considered acceptable. The model fit was checked using residual plots. Statistical significance was set at *p* < 0.05. Data are reported as means ± standard deviation.

## 3. Results

On the basis of data of maximal dorsiflexion ROM, all subjects were divided into three groups using cluster analysis (flexible: n = 7 [four male and three female subjects]; moderate: n = 22 [eight male and fourteen female subjects]; and tight: n = 12 [eight male and four female subjects]). All measured variables for each group are shown in Table 1.

### 3.1. Differences Between the Groups

Maximal dorsiflexion ROM exhibited a significant difference between the groups (*p* < 0.001). Post hoc testing showed that ROM in the flexible group was greater than that in the moderate (*p* < 0.001) and tight (*p* < 0.001) groups, and ROM in the moderate group was greater than that in the tight group (*p* < 0.001). However, there were no significant differences in age, height, weight, exercise time, and number of men and women between the groups (Table 1, all *p* > 0.08).

There was no significant difference in passive torque at −4° between the groups, whereas displacement of the MTJ showed a significant difference between the groups (*p* < 0.001). Post hoc testing showed that MTJ displacement in the tight group was greater than that in the moderate (*p* = 0.043) and flexible (*p* < 0.001) groups, and MTJ displacement in the moderate group was greater than that in the flexible group (Table 1, *p* = 0.036).

Stretch tolerance showed a significant difference between the groups (*p* < 0.001), although there was no significant difference in electromyography between the groups (*p* = 0.060). Post hoc testing showed that stretch tolerance in the flexible group was greater than that in the moderate (*p* = 0.003) and tight (*p* < 0.001) groups, and stretch tolerance in the moderate group was greater than that in the tight group (Table 1, *p* = 0.004).

No significant differences were observed in muscle thickness (*p* = 0.616), cross-sectional area (*p* = 0.850), pennation angle (*p* = 0.150), or fascicle length (*p* = 0.543) between the groups (Table 1).

Echo intensity showed no significant difference between the groups (Table 1, *p* = 0.067).

### 3.2. Correlation Coefficient

A significant correlation with maximal dorsiflexion ROM was found for passive torque at −4° (*r* = −0.335), displacement of MTJ (*r* = −0.659), and stretch tolerance (*r* = 0.784), but not for other variables (Table 2, all *p* > 0.09).

### 3.3. Stepwise Multiple Regression Analysis

**Figure 4:**
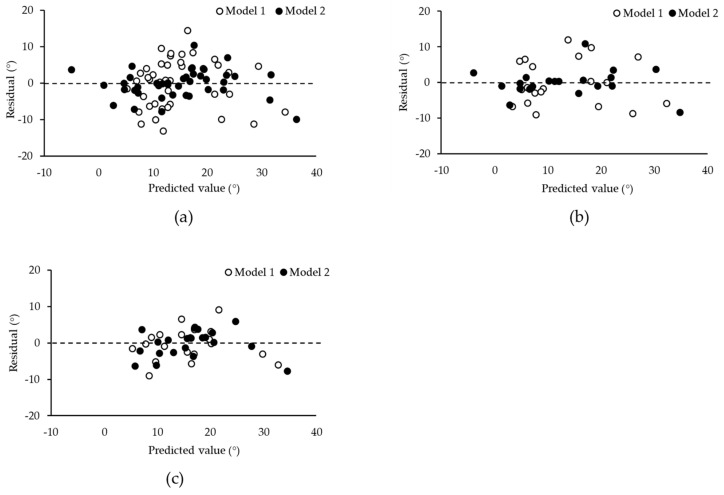
Residual plots of stepwise regression analysis for the (**a**) all-subjects model, (**b**) men-only model, and (**c**) women-only model. Predicted values of maximal dorsiflexion ROM were calculated using model 1 (open circles) and model 2 (closed circles).

The regression analyses for maximal dorsiflexion ROM, as shown in Table 3, included models for the all-subjects model, as well as the gender-specific models. Initially, stretch tolerance was included in model 1 for all models, and the addition of passive torque at −4° significantly improved the models. This improvement is reflected in the following adjusted *R*^2^ values: 0.832 for the all-subjects model, 0.853 for the men-only model, and 0.786 for the women-only model. The model fit was assessed using residual diagnostics to evaluate the independence and distributional properties of the residuals. The residual plots confirmed that there were no outliers in the residuals and that no specific trend was observed (Figure 4).

## 4. Discussion

The purpose of the current study was to determine the contributions of mechanical, neural, morphological, and muscle quality factors to individual differences in maximal dorsiflexion ROM. In addition, multiple regression analysis was used to determine the contribution of these factors to maximal dorsiflexion ROM. The results revealed that mechanical and neural factors were associated with individual differences in maximal dorsiflexion ROM, whereas morphological and muscle quality factors were not. To the best of our knowledge, this study is the first to examine the combined influences of mechanical, neural, morphological, and muscle quality factors on individual differences in maximal dorsiflexion ROM, incorporating multiple factors identified in previous studies.

### 4.1. Factors Detecting Maximal Dorsiflexion ROM

Stretch tolerance and passive torque at −4° were retained in all three regression models. Previous studies have reported the existence of sex-related effects on the displacement of the MTJ during ankle dorsiflexion and on the correlation between joint ROM and stretch tolerance [26,27]. Therefore, we tested men-only, women-only, and all-subjects models to examine sex differences in maximal dorsiflexion ROM. Interestingly, however, the selected explanatory variables were the same regardless of sex, and the adjusted *R*^2^ value did not differ substantially, revealing that stretch tolerance and passive torque at −4° explained approximately 80% of the maximal dorsiflexion ROM. Thus, no sex differences were found in the results of the multiple regression analysis with maximal dorsiflexion ROM as the dependent variable.

Stretch tolerance was a particularly influential factor for maximal dorsiflexion ROM (*β* = 0.826–0.856). Even when stretch tolerance was the only explanatory variable, the adjusted *R*^2^ value ranged from 0.474 to 0.663. Thus, stretch tolerance appeared to be the most important factor explaining individual differences in maximal dorsiflexion ROM. In contrast, MTJ displacement was not selected as a predictor, although there were significant group differences and a correlation with maximal dorsiflexion ROM. We speculate that although MTJ displacement is correlated with stretch tolerance, stretch tolerance is more strongly associated with maximal dorsiflexion ROM. This finding is in agreement with the results of previous studies [5,6], which indicated that stretch tolerance is an important factor in explaining individual differences in maximal joint ROM. However, other variables may play only a minimal role in predicting maximal dorsiflexion ROM. To our knowledge, no previous studies have examined the influence of each factor contributing to maximum dorsiflexion ROM using multiple regression analysis. Thus, no direct comparisons can be made between the current findings and those of previous studies as to whether these variables may play only a minimal role in predicting maximal dorsiflexion ROM.

Although stretching is commonly used to increase ROM, the current study suggests that stretching to increase stretch tolerance and decrease passive torque at −4° may effectively improve maximal dorsiflexion ROM. Previous studies have explained increased stretch tolerance and decreased passive torque at a given angle as mechanisms for improving ROM with static stretching, dynamic stretching, and proprioceptive neuromuscular facilitation stretching [15,23,24,28,29,30,31,32]. However, stretching has a limited effect in reducing the passive torque at a given angle, and the passive torque at less dorsiflexed angles, such as −4°, is relatively low to begin with. Therefore, further reduction in passive torque may be challenging [23,33]. In addition, a small number of previous studies have reported an increase in maximal joint ROM after stretching, only by increasing stretch tolerance without a decrease in passive torque [23,33,34,35]. Therefore, the current findings suggest that increasing stretch tolerance through stretching is the most important factor for improving maximal dorsiflexion ROM among mechanical, neural, morphological, and muscle quality factors.

The validity of the estimation of maximal dorsiflexion ROM and the estimation of change in maximal dorsiflexion ROM caused by stretching using the model equation developed in this study was determined on the basis of the results of previous studies [24,32,36,37]. To the best of our knowledge, no previous studies have reported passive torque at −4°. Thus, the values of maximal dorsiflexion ROM and the change in maximal dorsiflexion ROM before and after stretching were calculated by substituting the values of stretch tolerance reported in previous studies [24,32,36,37] into the equation for model 1 from all subjects in this study (Table 4). The level of agreement between the results of previous studies [32,36] using dynamic and static stretching and the values calculated by the model equation in the current study was relatively high, with errors in maximal dorsiflexion ROM ranging from 0.6° to 3.1° and errors in change in maximal dorsiflexion ROM by stretching ranging from 0.9° to 1.0°. However, when calculated on the basis of the results of other previous studies using static stretching [24,37], maximal dorsiflexion ROM exhibited a large error, ranging from 7° to 18.1°, while the error in the change in maximal dorsiflexion ROM by stretching was small, ranging from 0.9° to 1.1°. Errors in maximal dorsiflexion ROM were small between the present study and previous studies in which measurements were taken in the sitting position, as in the present study [32,36]. However, errors were large between the present study and previous studies in which measurements were taken in the prone position [24,37]. A comparison of these previous studies using different measurement postures shows differences in maximal dorsiflexion ROM despite similar levels of stretch tolerance and, conversely, differences in stretch tolerance despite similar maximal dorsiflexion ROM [24,32,36,37]. Therefore, differences in measurement posture may have contributed to the large differences in maximal dorsiflexion ROM errors, depending on the previous studies used to calculate the maximal dorsiflexion ROM [24,32,36,37]. However, it should be noted that the estimation error of the change in maximal dorsiflexion ROM was small regardless of the measurement posture. Consequently, we propose that the estimation equation developed in this study may be helpful for predicting the amount of change in maximal dorsiflexion ROM with a low level of error, regardless of the measurement posture, if the change in stretch tolerance caused by an intervention can be estimated. The current data indicate that stretch tolerance has not been overemphasized in this study as a primary determinant of maximal dorsiflexion ROM and was the most important determinant of maximal dorsiflexion ROM.

### 4.2. Differences in Mechanical, Neural, Morphological, and Muscle Quality Factors Between Groups and Correlation Coefficients with Maximal Dorsiflexion ROM

MTJ displacement in mechanical factors was significantly different among the three groups (Table 1). The current results revealed that MTJ displacement was greatest in the tight group, and there was a significant negative correlation between maximal dorsiflexion ROM and MTJ displacement. Thus, the findings suggested that the greater the maximal dorsiflexion ROM, the smaller the MTJ displacement (i.e., the smaller the change in muscle lengthening) for an increase in dorsiflexion angle during the last 13°. Blazevich et al. [5] reported that from −20° to 20° of dorsiflexion, muscle and tendon lengthening increased linearly with increasing dorsiflexion, but from 20° onward, muscle lengthening became negligible, and tendon lengthening became greater. In the present study, the average maximal dorsiflexion ROM of the tight and moderate groups was less than 20°, suggesting a linear increase in muscle lengthening with increasing dorsiflexion, resulting in greater MTJ displacement. By contrast, the flexible group had an average maximal dorsiflexion ROM greater than 20°, suggesting less muscle lengthening, resulting in smaller MTJ displacement. However, because the increase in muscle–tendon unit length with ankle dorsiflexion is mostly caused by an increase in either or both muscle or tendon length [24], we speculate that the contribution of the tendon length change was greater than that of the muscle length change in the increase in dorsiflexion angle in the last 13° in the flexible group. This speculation is supported by a previous study reporting that the greater maximal dorsiflexion ROM group exhibited greater tendon lengthening at the maximal dorsiflexion position than the lesser maximal dorsiflexion ROM group [38]. Thus, the current findings suggest that, in individuals with greater maximal dorsiflexion ROM, the length change associated with dorsiflexion of the tendon rather than the muscle is related to the determination of individual maximal dorsiflexion ROM, whereas, in individuals with less maximal dorsiflexion ROM, the length change in both the muscle and tendon is related to the determination of individual maximal dorsiflexion ROM.

The results revealed group differences in the stretch tolerance of neural factors and a significant positive correlation between maximal dorsiflexion ROM and stretch tolerance (Table 1 and Table 2). The findings of two previous studies are in accord with the current results [5,6], suggesting that stretch tolerance is an important factor in explaining individual differences in maximal joint ROM. However, Moltubakk et al. [7] compared maximal dorsiflexion ROM and stretch tolerance between ballet dancers and non-ballet dancers, and they reported that maximal dorsiflexion ROM was greater in the ballet dancer group, whereas there was no difference in stretch tolerance between the groups. In athletes such as ballet dancers or gymnasts, for whom gaining greater maximal joint ROM significantly impacts their competitive performance [3], flexibility training such as stretching is often implemented from an early age [39]. By contrast, none of the subjects in the current study habitually stretched their ankle joints. Therefore, the factors explaining maximal joint ROM may differ between groups with specific athletic training, such as dancers and gymnasts, and populations who have not undertaken specific athletic training, as in the current study. The mechanisms underlying stretch tolerance are not fully understood but have been suggested to be related to stretch feedback, pressure feedback, pain feedback, or supraspinal registration of these signals [5]. Therefore, detailed clarification of the mechanisms underlying stretch tolerance and its modulation methods in future studies may lead to a better understanding of the mechanisms related to individual differences in maximal joint ROM and the establishment of effective methods for increasing maximal joint ROM.

Regarding mechanical and neural factors, we found no group differences in passive torque at −4° and electromyography amplitude. Previous studies reported that subjects with greater maximal dorsiflexion ROM exhibited lower passive torque at a given joint angle [5,7]. In the current study, passive dorsiflexion was started at −30°, and the maximal dorsiflexion angle achieved by any of the subjects was −4°. Thus, the comparison of passive torque at a given joint angle was conducted at −4°. Passive torque increases with increasing dorsiflexion angle, but the passive torque produced was negligible in the plantar flexion position or when the dorsiflexion angle was small [23,24,33,40]. A comparison of passive torque between the greater and lesser maximal dorsiflexion ROM groups in a previous study revealed a difference in passive torque at 30°, but no significant difference was observed at angles less than 30° [5]. Therefore, it is likely that no significant difference was found in the present study comparing passive torque at −4°. However, a significant negative correlation was found between maximal dorsiflexion ROM and passive torque at −4° (Table 2), indicating that although there were no group differences, there was a relationship whereby subjects with greater maximal dorsiflexion ROM exhibited less passive torque at −4°.

Results regarding differences in electromyography responses in greater and lesser maximal dorsiflexion ROM groups have differed among previous studies [5,7]. One study that examined a sample of the general population reported no group differences in electromyography responses during passive dorsiflexion [5], which is similar to the findings of the present study. This previous study suggested that less involuntary muscle activity during dorsiflexion in individuals with greater maximal dorsiflexion ROM contributes to less passive torque at a given angle [5]. However, in the present study, there were no group differences in electromyography, suggesting that factors other than electromyography may have contributed to the relationship in which subjects with greater maximal dorsiflexion ROM exhibited less passive torque at −4°.

Morphological factors did not differ between groups, and there was no significant correlation between morphological factors and maximal dorsiflexion ROM. No group differences in pennation angle and cross-sectional area were found in the present study, which is consistent with the findings of several previous studies [5,6,7]. However, the results regarding muscle thickness and fascicle length in our study differed from the findings of two previous studies [7,8]. Moltubakk et al. [7] reported that dancers exhibited greater muscle thickness and fascicle length in addition to greater maximal dorsiflexion ROM compared with controls. Additionally, Suga et al. [8] reported that muscle thickness was negatively correlated with maximal dorsiflexion ROM. One possible reason for the discrepancy between these results and those of the present study is the influence of subject characteristics. This may have occurred because the subjects in the previous study were adapted to dance training [7], and the range of maximal dorsiflexion ROM of the subjects was very different from that of the subjects in the present study (present study, from −3.5 to 34.4 vs. previous study, from 20.0 to 47.0) [8]. Therefore, the current findings suggested that there was no significant correlation between maximal dorsiflexion ROM and morphological factors in the general population, which exhibits a wide range of maximal dorsiflexion ROM.

Echo intensity in the gastrocnemius muscle did not differ between groups and was not significantly correlated with maximal dorsiflexion ROM. Koutedakis et al. [14] reported that muscle factors contributed approximately 10% to joint maximal ROM as limiting factors. The authors also mentioned that muscle factors include connective tissue and muscle fat concentration [14]. In addition, Morse et al. [24] reported that the amount of MTJ displacement associated with dorsiflexion after static stretching could not be explained by changes in muscle fascicle length alone. The authors suggested that connective tissue changes may have affected the amount of MTJ displacement associated with dorsiflexion after static stretching [24]. Thus, we expected to find a significant relationship between maximal dorsiflexion ROM and muscle quality factors, but no such significant relationship was found. Thus, the current findings indicated that muscle quality did not contribute as a factor to maximal dorsiflexion ROM, or if it did, the contribution was very limited.

The subject’s posture in passive-dorsiflexion measurement may influence the determinants of maximal dorsiflexion ROM. Maximal dorsiflexion ROM has been reported to be affected by flexion or extension of the knee or hip, and has been reported to vary by approximately 30° [41]. Maximal dorsiflexion ROM was 22.3° smaller in the sitting posture with the hip at 90° and knee in full extension than in the supine posture with the hip and knee in full extension, and maximal dorsiflexion ROM was 30.8° smaller in the supine posture with the hip and knee at 90° flexion [41]. In addition, maximal dorsiflexion ROM was 17.7° lower in the sitting posture with the hip at 90° and the knee in full extension than in the sitting posture with the hip at 150° and the knee in full extension [42]. These changes have previously been explained as being caused by changes in the transmission of tension through the fascia and changes in the tension in the sciatic nerve tract in relation to hip flexion and knee extension [42,43]. However, no change was reported in the passive torque–angle curve or shear elastic modulus when the maximal dorsiflexion ROM decreased with hip flexion [42]. Thus, changes in maximal dorsiflexion ROM with hip flexion are primarily caused by changes in tension in the sciatic nerve tract. A previous study reported that trunk flexion causes movement of the sciatic nerve [44]. On the other hand, maximal dorsiflexion ROM was 8.47° greater in the supine position with the hip and knee 90° flexed than in the supine position with the hip and knee in full extension, suggesting that knee flexion has a greater effect on maximal dorsiflexion ROM than hip flexion [41]. Because changes in maximal dorsiflexion ROM within an individual caused by such changes in measurement posture may affect the factors contributing to the difference in maximal dorsiflexion ROM between individuals, it may be useful to evaluate the determinants of maximal dorsiflexion ROM in a different posture from that used in the present study.

The current study involved the limitation that only the current exercise habits of the subjects were measured, whereas their previous exercise history (including frequency, intensity, and duration) was not measured. Maximal dorsiflexion ROM may be influenced by all previous exercise experiences of the subject. Thus, future studies should examine previous exercise experience.

## 5. Conclusions

The current findings clarified that mechanical and neural factors, but not morphological and muscle quality factors, are involved in individual differences in maximal dorsiflexion ROM, and that the contribution of stretch tolerance from neural factors is particularly high. Therefore, it is particularly important to increase stretch tolerance to increase maximal dorsiflexion ROM. If the results of this study can be extended to establish a technique to maximize the improvement of stretch tolerance in the future, an effective improvement in maximal dorsiflexion ROM can be expected. As a result, it may be possible to improve athletic performance through increased flexibility, prevent injury during exercise, and improve the gait and balance of older people more efficiently.

## Figures and Tables

**Table 1:** Differences in the physical description, mechanical, neural, morphological, and muscle quality factors among the flexible, moderate, and tight groups.

		Group 1 (Flexible)	Group 2 (Moderate)	Group 3 (Tight)
	Number of subjects	7 (M = 4, F = 3)	22 (M = 8, F = 14)	12 (M = 8, F = 4)
	Maximal dorsiflexion ROM (°)	28.4 ± 3.0 *^,†^	16.4 ± 4.6	3.1 ± 3.5
	Age (year)	21.1 ± 1.2	19.9 ± 1.4	20.1 ± 1.2
	Height (cm)	171.5 ± 12.0	162.5 ± 10.1	167.9 ± 7.6
	Body weight (kg)	64.0 ± 13.4	58.6 ± 17.7	57.9 ± 14.5
	Exercise time (min)	97.9 ± 186.6	194.1 ± 183.1	135.0 ± 125.7
Mechanical factors				
	Passive torque at −4° (Nm)	4.4 ± 1.6	4.4 ± 1.9	6.0 ± 2.3
	MTJ displacement (mm)	3.5 ± 1.2 ^†^	5.0 ± 1.4	6.2 ± 1.4
Neural factors				
	Stretch tolerance (Nm)	29.1 ± 9.2 ^†^	16.1 ± 7.8	9.1 ± 3.6
	Electromyography (%MVC)	1.0 ± 0.7	1.0 ± 1.3	1.8 ± 2.3
Morphological factors				
	Muscle thickness	18.1 ± 1.8	17.9 ± 2.9	18.4 ± 2.8
	Cross-sectional area (cm^2^)	15.1 ± 3.1	16.3 ± 4.8	16.1 ± 5.8
	Pennation angle (°)	16.0 ± 2.4	16.2 ± 2.6	18.0 ± 3.1
	Fascicle length (mm)	64.4 ± 8.0	60.1 ± 8.2	61.5 ± 10.1
Muscle quality factor				
	Echo intensity (a. u.)	73.4 ± 14.2	74.1 ± 10.5	65.1 ± 8.8

* Represents a significant difference with Group 2. ^†^ Represents a significant difference with Group 3. Abbreviations: MTJ, muscle–tendon junction; MVC, maximal voluntary contraction; and ROM, range of motion.

**Table 2:** Correlation coefficient between maximal dorsiflexion ROM and the physical description, mechanical, neural, morphological, and muscle quality factors.

		Correlation Coefficient with ROM (All Subjects)
	Age (year)	0.196
	Height (cm)	0.004
	Body weight (kg)	0.111
	Exercise time (min)	−0.067
Mechanical factors		
	Passive torque at −4° (Nm)	−0.335 *
	MTJ displacement (mm)	−0.659 *
Neural factors		
	Stretch tolerance (Nm)	0.784 *
	Electromyography (%MVC)	−0.305
Morphological factors		
	Muscle thickness	0.020
	Cross-sectional area (cm^2^)	−0.043
	Pennation angle (°)	−0.182
	Fascicle length (mm)	0.037
Muscle quality factor		
	Echo intensity (a. u.)	0.269

* Represents a significant correlation with ROM.

**Table 3:** Stepwise regression analysis as a dependent variable of the maximal dorsiflexion ROM.

Object		Independent Variables	B	*β*	Adjusted *R*^2^	*p*
All subjects						
	Model 1	Stretch tolerance	0.698	0.698	0.474	<0.001
		Constant	3.223			
	Model 2	Stretch tolerance	0.856	0.856	0.832	<0.001
		Passive torque at −4°	−2.827	−0.616		
		Constant	14.490			
Men						
	Model 1	Stretch tolerance	0.763	0.800	0.620	<0.001
		Constant	−1.675			
	Model 2	Stretch tolerance	0.814	0.853	0.853	<0.001
		Passive torque at −4°	−2.576	−0.481		
		Constant	13.361			
Women						
	Model 1	Stretch tolerance	1.028	0.825	0.663	<0.001
		Constant	2.320			
	Model 2	Stretch tolerance	1.030	0.826	0.786	<0.001
		Passive torque at −4°	−2.660	−0.357		
		Constant	11.897			

Independent variables were passive torque at −4°, muscle–tendon junction displacement, stretch tolerance, electromyography, muscle thickness, pennation angle, fascicle length, and echo intensity. Abbreviations: B, non-standardizing coefficient; *β*, standardized regression coefficient; *R*^2^, coefficient of determination; and ROM, range of motion.

**Table 4:** Stepwise regression analysis as a dependent variable of the maximal dorsiflexion ROM.

		Maximal Dorsiflexion ROM Before Stretching	Maximal Dorsiflexion ROM After Stretching	Change in ROM by Stretching
Static stretching				
	Mizuno [36]	14.6 (17.7)	18.2 (20.3)	3.6 (2.6)
	Morse et al. [24]	28.1 (35.1)	32.7 (40.6)	4.6 (5.5)
	Nakamura et al. [37]	36.6 (19.6)	41.8 (23.7)	5.2 (4.1)
Dynamic stretching				
	Mizuno [32]	20.6 (21.5)	23.8 (24.4)	3.2 (2.9)

Figures outside the brackets are data reported in previous studies; figures inside the brackets are data calculated from stretch tolerance data reported in previous studies using the model 1 equation developed in this study (maximal dorsiflexion ROM [°] = 0.698 × stretch tolerance [Nm] + 3.223). Abbreviations: ROM, range of motion.

## Data Availability

The data that support the findings of this study are available from the corresponding author upon reasonable request.
